# Perceived Hearing Loss and Availability of Audiologists in Appalachia

**DOI:** 10.13023/jah.0304.04

**Published:** 2021-10-25

**Authors:** Charles Pudrith AuD, Ellyn Grider, Blythe Kitner AuD

**Affiliations:** School of Allied Health and Communicative Disorders, Northern Illinois University, DeKalb IL

**Keywords:** Appalachia, hearing loss, rural health care, barriers to healthcare access

## Abstract

**Introduction:**

There is a high demand for audiologists throughout the United States. Previous research has supported an additional demand for these providers within Appalachia.

**Purpose:**

The purpose of the study was to determine if Appalachia has a disproportionally high demand for audiologists compared to the rest of the United States.

**Methods:**

A cross-sectional retrospective study was performed with population data from the Appalachian Regional Commission, the American Academy of Audiology, and the United States Census Bureau. County-level population-weighted averages of individuals with perceived hearing loss and number of audiologists per capita were compared between Appalachian and non-Appalachian counties.

**Results:**

A mean weighted 5.76 % of individuals reported hearing loss within Appalachia, which was 1.1% higher than the rest of the United States. The 1.14 audiologists per 100,000 individuals in Appalachian counties was not significantly lower than the 1.32 audiologists per 100,000 individuals found in non-Appalachian counties. Audiologists per capita decreased with increases in Beale code and percent reporting hearing loss.

**Conclusion:**

The high number of individuals reporting hearing loss supports an increased demand for audiologists in rural Appalachia. More research is needed to determine how to meet this demand or improve the efficacy of the limited number of providers.

## INTRODUCTION

Approximately 21.7% of U.S. adults are affected by hearing loss, which negatively impacts oral communication.^1^,^2^ Hearing loss restricts audibility of soft sounds and degrades the quality of louder sounds, thereby taxing the listener’s cognitive resources during conversation.^2^ Hearing loss leads to a withdrawal of activities and participation and reduces the quality of life.^2^,^3^ Those who reported difficulty with hearing scored more than twice as high on depression scales.^1^

Hearing loss treatment has been shown to increase activity participation and overall quality of life.^4^,^5^ Age-related and noise-induced hearing loss may be treated by audiologists or hearing aid dispensers. These types of hearing loss are typically treated with hearing aids, which are not covered by Medicare. Medicaid coverage of hearing aids varies among the states. Hearing loss with a medical pathology is first treated by an otolaryngologist. Once the otolaryngologist has determined that medical intervention will not restore hearing, then the patient is referred to an audiologist or hearing aid dispenser.

Audiologists are the only healthcare provider specially trained to diagnose the site of lesion of a hearing loss within the ear or along the neural auditory pathway, identify the need for referrals through advanced diagnostic testing, select and program hearing aids and implantable devices, and provide aural rehabilitation.^6^ Audiologists also diagnose and treat tinnitus, hyperacusis, auditory processing disorder, and balance disorders of the ear. In contrast, hearing aid dispensers focus on basic diagnostics and hearing aid selection and fitting.

The availability of audiologists is a problem throughout the United States.^7^ About two-thirds of the adult U.S. population reported that they have not had their hearing tested within the last ten years, and only half of those 65 and over have reported having a hearing test in the last five years.^8^,^9^ Regarding hearing aid use, only 3.7% of those who reported hearing problems indicated that they wore hearing aids.^9^ Reduced access is partly caused by the ‘inverse care law’ where there is a decrease of audiologists found in counties with an increase of reported hearing loss.^7^ This inverted relationship may be caused by the limitations in insurance coverage that force audiologists to work in healthy, affluent regions that can pay for their services out of pocket.^10^ Unfortunately, the shortage of audiologists, particularly in underserved regions, is expected to grow considerably when factoring in the increasing number of individuals over 65 over the next few decades. The problem is made worse by the fact that more audiologists are leaving the profession than entering the profession each year.^11^

Those who live in rural areas have a high demand for hearing health care, which is caused by both a decrease in access to healthcare providers and an increase in hearing loss.^12^,^13^ A recent study found that only half of those living in a rural area had access to a hearing healthcare provider.^14^ The reduced access was primarily caused by the increased driving distance.^14^,^15^ However, financial constraints and a lack of awareness were also causative factors.^16^ Increased hearing loss in rural areas is at least partially explained by occupational and recreational noise exposure. Occupationally, many individuals living in rural areas work in farming, which requires loud machinery.^17^ Recreationally, those who live in rural areas often participate in loud activities, including firearm and motor vehicle use and tractor pulls.^18^,^19^ Additionally, many individuals living in rural areas have shown a reluctance to participate in hearing conservation programs.^20^

Appalachia is a primarily rural region in the U.S. that may have a high demand for adult audiological services. As found in many rural regions, adults living in Appalachia have reported that both the cost of audiological services and the distance to the nearest provider made seeking treatment prohibitive.^21^ Barriers have also been identified when seeking audiological care for children in Appalachia.^22^ These barriers include poor communication of results, lack of local resources, insurance-related delays, and conflicts with family and work responsibilities. Regarding the need for adult audiological services, Appalachia is home to many retired coal miners who likely have higher incidences of noise-induced hearing loss. In 1990, only 40% of coal miners exposed to hazardous noise levels reported wearing hearing protection.^23^ The noise exposure traditionally found in rural areas combined with noise exposure from coal mining may make Appalachians especially at risk for high levels of hearing loss. Previous studies have shown that Appalachians have reduced access to medical healthcare providers compared to non-Appalachian rural regions.^24^

Previous studies have supported a lack of access to hearing healthcare in Appalachia. The primary purpose of this study is to compare the demand for adult audiological services in Appalachia to the demand of audiological services throughout the rest of the country. Demand will be evaluated by measuring the percent of individuals reporting hearing loss and the count of audiologists per capita. Additionally, this study is designed to determine how rurality may play a role in this demand. Identifying an Appalachian-specific disparity in access to audiologists may support expansion of access to hearing services provided to this area.

## METHODS

### Retrospective Data Collection

Percentage of reported hearing loss, number of audiologists registered with the American Academy of Audiology (AAA), Beale codes, and county level classifications throughout the contiguous U.S. were downloaded from publicly available databases within five organizations: (1) U.S. Census Bureau, (2) American Academy of Audiology, (3) U.S. Department of Agriculture, (4) Appalachian Regional Commission, and (5) National Oceanic and Atmospheric Administration. The data collected from these organizations were used to evaluate the demand for audiological services in Appalachia compared to the rest of the contiguous U.S.

All counties in the contiguous U.S. were placed into one of ten regional groups to measure the demand for audiological services in Appalachia compared with other geographical regions (Table 1). To accomplish this goal, data was first downloaded from the Appalachian Regional Commission to identify and label Appalachian counties.^25^ Most states that contained Appalachian counties were split between counties within and outside of Appalachia. After labeling the Appalachian counties, data from the National Oceanic and Atmospheric Administration were used to classify all remaining counties in the contiguous U.S. into nine other regions, bringing the total number of regions to ten.^26^ The counties that were originally classified as Appalachian were not changed so that every county was classified into only one region.

The total population for each county was downloaded from the U.S. Census Bureau, which sends out a survey, either by mail or by visit, to every home in America every ten years. The results of this survey were accessed using the U.S. Census Bureau data exploration tool.^27^ Population data were used to weigh each county to compare county-level data among regions with counties serving as individual samples.

The demand for audiological services was evaluated by first measuring the estimated percentage of individuals reporting hearing loss in each county. These measurements were obtained from the American Community Survey. This survey is sent out through the internet, mail, or phone interview to approximately 10% of homes in the United States.^28^ To ensure that the data was a representation of the national population, the sample selection was weighted based on housing, rurality, race, age, sex, geography. Additionally, coverage rates were adjusted to reduce over or under-sampling of specific groups.^29^ One question that the American Community Survey asks participants was if they have difficulty hearing. The aggregated percent of individuals reporting a hearing loss for every county in the U.S. is publicly available on the U.S. Census Bureau data exploration tool.^27^ The data downloaded for this study were collected between 2013 and 2018.

The demand for audiological services was further evaluated by tallying AAA registered audiologists per 100,000 individuals in each county. The number of AAA registered audiologists was obtained through email correspondence with the AAA, the largest organization of audiologists in the United States.^30^ There are some practicing audiologists that are not registered with AAA. Unfortunately, these audiologists could not be included because it is difficult to determine which audiologists are registered under multiple organizations.

Beale Codes (also called Rural–urban continuum codes) have been assigned to each county by the U.S. Department of Agriculture.^31^ These codes are based on the population of each county and their approximation to counties with higher populations. The values range from one to nine, with one indicating the most urban environment and nine indicating the most rural environment. These values were included to determine if rurality explained differences in the demand for Audiologists across regions.

## ANALYSES

All analyses were conducted with population-weighted county-level data compared between Appalachian and non-Appalachian regions across the ten geographical regions previously described. All data were averaged within regions or across non-Appalachian regions to reduce the effects of sampling variability caused by the American Community Survey, which only sampled 10% of the population. Also, comparing means across regions reduced the effects of sampling bias because biases likely equally affect both Appalachian and non-Appalachian counties. These comparisons cancel out their effects. For all tests, alpha values were set to 0.001 to account for multiple tests based on a Bonferroni correction. All analyses were conducted using SPSS (Armonk NY: IBM Corp).

To evaluate the demand for audiological services in Appalachia, percent of individuals reporting hearing loss, the number of AAA registered audiologists per 100,000 individuals, and Beale codes were compared between Appalachian and non-Appalachian regions. First, the percent of individuals reporting hearing loss, number of AAA registered audiologists per 100,000 individuals and Beale codes were compared between Appalachian and non-Appalachian counties using a three separate t-tests. Then, the percent of individuals reporting hearing loss, number of AAA registered audiologists per 100,000 individuals, and Beale codes for each county were compared between Appalachia and the other nine regions using three separate post hoc analyses of variance.

Linear regression analyses were used to evaluate the relationship of county-specific variables, without considering region. The association between the pairwise combinations of the percent of individuals reporting hearing loss, the number of AAA registered audiologists per 100,000 individuals, and the Beale code was assessed across all counties in the contiguous United States.

Two multiple linear regression analyses were used to assess the combined relationship of the region (Appalachia vs. non-Appalachia), the percent of individuals reporting hearing loss, the number of AAA registered audiologists per 100,000 individuals, and Beale codes. The first multiple linear regression analysis was used to assess the effect of Beale code and region on the percentage of individuals reporting hearing loss. This analysis was performed to determine if Beale codes were associated with individuals reporting hearing loss, after adjusting for region, and to determine if regions were associated with percent of individuals reporting hearing loss, after adjusting for Beale codes. To complete the regression analyses, counties were set to 0 for non-Appalachian counties and 1 for Appalachian counties. Then, a second multiple linear regression analysis was used to measure the effect of Beale codes and percent of individuals reporting hearing loss on the number of AAA registered audiologists per 100,000 individuals. This second analysis was performed to determine if Beale codes were associated with the number of audiologists per 100,000 individuals, after adjusting for the percent of individuals reporting hearing loss and determine if the percent of individuals reporting hearing loss and the number of audiologists per 100,000 individuals were associated, after adjusting for Beale codes.

## RESULTS

The mean population-weighted percent of individuals reporting hearing loss was compared between Appalachian and non-Appalachian counties. The mean was found to be 5.76 percent in Appalachia, which was significantly (p<0.001) higher than the 4.66 percent found throughout the rest of the country (Table 2). When the mean population-weighted percent of individuals reporting hearing loss in Appalachia was compared to the mean in specific regions, mean in Appalachia was significantly (p<0.001) higher than the means found in the Northeast, Southeast, Ohio Valley, South, Upper Midwest, and West.

The mean population-weighted count of audiologists per 100,000 individuals was compared between Appalachian and non-Appalachian counties. This mean was 1.14 for Appalachian counties, which was not significantly less than the 1.32 found in non-Appalachian counties (Table 2). The mean population-weighted count of audiologists per 100,000 individuals in Appalachia was significantly (p < 0.001) less than the 2.17 found in the northeast. There were no other significant differences between Appalachian counties and any of the other regions.

Mean population-weighted Beale codes were also compared between Appalachian and non-Appalachian counties. This mean was 4.54 for Appalachian counties, which was not significantly different from the 4.07 found in non-Appalachian counties (Table 2). However, when compared with specific regions, the mean population-weighted Beale code for Appalachia was significantly higher than the means for the Northeast and the West, and lower than the means for the Upper Midwest and the Northern Rockies.

**Figure 1:**
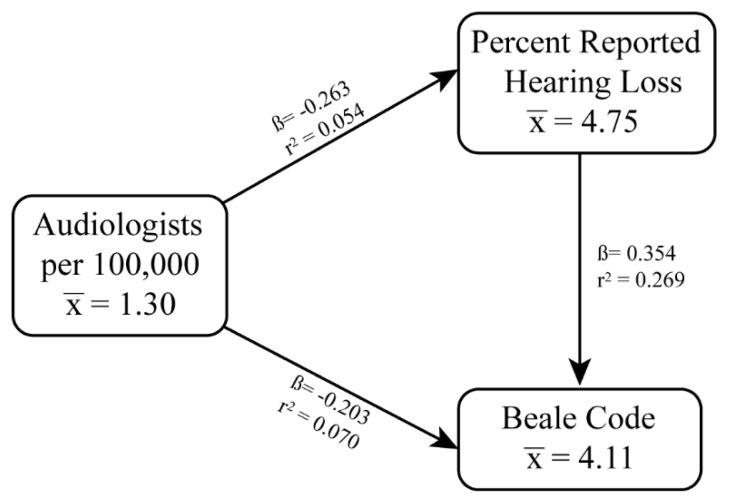
Weighted means, Beta coefficients, and r^2^ values of linear regression models between percent reported hearing loss, Audiologists per 100,000, and Beale codes. All associations were statistically significant (p<0.001).

Linear regression analyses were used to measure associations between the three pairwise comparisons among (1) the percent with reported hearing loss, (2) the number audiologists per 100,000 individuals, and (3) Beale codes (Figure 1). Again, population-weighted county-level means were used for these analyses, and regions were not included. All three comparisons were statistically significant (p<0.001). The analysis revealed that for every one percent increase of individuals reporting hearing loss, the number of audiologists per 100,000 individuals decreased by 0.263. For every unit increase in the Beale code, the number of audiologists per 100,000 individuals decreased by 0.203. Also, for every unit increase in the Beale code, the percent of individuals reporting hearing loss increased by 0.354. Collectively, these results indicate that decreases in audiologists per 100,000 individuals are explained by increases in both percentage of individuals reporting hearing loss and Beale code.

Two multiple linear regression analyses were used to assess the combined relationship of region (Appalachia vs. non-Appalachia), the percent of individuals reporting hearing loss, the number of AAA registered audiologists per 100,000 individuals, and the Beale code. First, the effect of Beale code and Appalachian classification on the percent reported hearing loss was measured. Both Beale code and Appalachian classification were significantly and independently associated with percent reported hearing loss (Table 3). Adjusting for Beale code did not affect the association between the percent of individuals reported hearing loss and Appalachian classification. Also, adjusting for Appalachian classification did not affect the association between Beale codes and the percent of individuals reported hearing loss.

In the second analysis, the effect of Beale codes and percent reported hearing loss on the count of audiologists per 100,000 individuals was evaluated. Both

Beale codes and the percent reported hearing loss were significantly and independently associated with the count of audiologists per 100,000 individuals. Adjusting for the Beale code did not affect the association between the count of audiologists per 100,000 individuals and percent reported hearing loss and adjusting for percent reported hearing loss did not affect the association between Beale codes and the count of audiologists per 100,000 individuals.

## IMPLICATIONS

An estimated 5.76% of Appalachians reported hearing loss, which is about 1.1% higher than non-Appalachian regions. The estimated number of audiologists per 100,000 individuals in Appalachia was 1.14, which was not significantly lower than the 1.32 found in non-Appalachian counties. These findings collectively indicate that rural Appalachians have an increased demand for audiological services compared to the rest of the country. Previous studies demonstrating a lack of access to hearing care providers in Appalachia likely found the greatest access restriction in the more rural counties.^21^,^22^ Additionally, our findings support a previous study that found that as the Beale code and number of individuals reporting hearing loss increase, the number of audiologists per capita decreases.^7^

A multilinear regression model showed that those from Appalachia had more hearing loss than those from non-Appalachian counties, even after accounting for differences in Beale code. These findings were further supported with region-specific analyses which showed that overall, there was a higher percent of reported hearing loss in Appalachia compared to many regions, regardless of each region’s Beale code relative to Appalachia. The Appalachian-specific hearing loss may have been caused by the Appalachian coal miner’s reluctance to wear hearing protection in the 1990s.^23^ The combination of hearing loss traditionally found in rural areas with Appalachian-specific hearing loss may drive up the demand for Audiologists in this region.^12^,^13^,^18^–^20^

Audiologists are struggling to fill the need for hearing health care. Also, the future of audiology is projected to worsen as the age of the population increases and the overall number of audiologists is expected to decrease.^7^ This decrease may shift hearing rehabilitation to hearing aid dispensers, who are not trained to run advanced diagnostic testing, identify the need for medical referral, or perform aural rehabilitation. Additionally, audiologists are the only healthcare practitioners trained to fit implantable devices, treat tinnitus and hyperacusis, and diagnose auditory processing disorder and balance function.^6^ The results of this study show that rural Appalachia may struggle more than the rest of the country to obtain these services. Changes should be addressed in these regions to fulfill this demand.

Appalachia may benefit from motivating audiologists to work in this region, as well as an increased partnership with other healthcare providers. Incentives, such as student loan forgives, have motivated other healthcare professions to relocate to other rural areas.^32^ Given the high cost of a 4-year graduate audiology degree, offering student loan forgiveness may motivate young audiologists to practice in rural Appalachia. Other financial incentives may include changing insurance coverage so that audiologists can bill for more of their services.^14^ Additionally, including rural placements within audiology education programs may increase participation. These placements were found to be a significant factor in predicting participation in rural health care for other healthcare providers.^32^

Appalachia may also benefit from new service delivery models that use eHealth technology and either community healthcare workers or audiology assistants to increase affordability.^33^ Electronic health may improve audiological services in rural areas because mobile networks now cover 99% of North America.^34^ Providing community health workers or audiology assistants with smartphones and calibrated transducers may expand services to those who cannot afford audiological services through traditional methods check.^35^ Using community healthcare workers in other areas of health has been successful, which supports their utility in audiology.^36^

Increasing cooperation between audiologists and physicians may also improve hearing care in rural Appalachia. Physician advocacy was a major factor predicting patient’s history of receiving a hearing test.^9^ In addition, audiologists may benefit from providing courses for continuing education units to physicians that teach them the importance of hearing health care.^14^

Limitations of this study include estimates based on sample size. Reported hearing loss was estimated from surveys that were only distributed to 10% of the population, and audiologists per capita were estimated from the count of audiologists registered with the American Academy of Audiology and population data.^28^,^30^ Audiologists registered from other organizations could not be included because it is difficult to determine which audiologists were registered with multiple organizations. The effects of these limitations were reduced by the study design, which compared values across regions. However, it is possible that the effects of these limitations were unequal across regions. Additional limitations include the lack of specificity in reported hearing loss. The American Community Survey only used a yes/no question and was not able to account for the severity of the loss.

Appalachians have more reported hearing loss than the rest of the contiguous united states, even after adjusting for rurality. There is an overall shortage of audiologists, which increases with rurality. Combined, these findings indicate that there is a demand for improvements in audiological services in rural Appalachia. These improvements may include incentivizing audiologists to work in Appalachia, utilizing electronic health care, or improved cooperation between audiologists and physicians in this region. More work is needed to evaluate the efficacy of these interventions.

**Summary Box**

**What is already known about this topic?** Appalachians have reduced access to health care compared to the rest of the country and a history of noise exposure.

**What is added by this report?** This study found that Appalachians have an increased number of individuals reporting hearing loss compared to the rest of the country and may have fewer audiologists.

**What are the implications for future research?** More work is needed to determine where in Appalachia the demand for audiological care is highest so that changes can be made to support the needs of the communities.

## Figures and Tables

**Table 1:** Count of counties and division of states among ten regions

Region	Number of Counties	States
Appalachia	422	NY*, PA*, WV, OH*, MD*, VA*, KY*, NC*, TN*, SC*, GA*, AL*, MS*
Northeast	170	ME, NH, VT, NY*, PA*, MA, RI, CT, NJ, DE, MD*
Southeast	402	VA*, NC*, SC*, AL*, GA*, FL
Ohio Valley	475	MO, IL, IN, OH*, KY, TN*
South	633	KS, OK, TX, AR, LA, MS*
Upper Midwest	341	MN, WI, IA, MI
Northern Rockies	291	MT, ND, SD, WY, NE
Southwest	141	AZ, UT, CO, NM
West	75	CA, NM
Northwest	119	WA, OR, ID

* States that have counties in Appalachia and one non-Appalachian region

**Table 2:** Population-weighted mean (standard error) of percent reported hearing loss, AAA registered audiologists per 100,000 individuals, and Beale codes for values averaged within 10 regions and all non-Appalachian counties. P-values represent comparisons to weighted means within Appalachia.

	Percent Reported Hearing Loss		AAA registered audiologists per 100,000		Beale Code	
	Wt Mean (SE)	P-Value	Wt Mean (SE)	P-Value	Wt Mean (SE)	P-Value
**Appalachia**	5.76 (0.11)	–	1.14 (0.12)	–	4.54 (0.16)	–
**Non-Appalachia**	4.66 (0.03)	< 0.001*	1.32 (0.04)	0.159	4.07 (0.05)	0.005
**Northeast**	3.63 (0.07)	< 0.001*	2.17 (0.09)	< 0.001*	2.65 (0.10)	< 0.001*
**Southeast**	4.66 (0.08)	< 0.001*	0.81 (0.09)	1.00	3.74 (0.11)	0.001
**Ohio Valley**	4.89 (0.08)	< 0.001*	1.51 (0.10)	0.716	4.43 (0.12)	1.00
**South**	5.10 (0.08)	< 0.001*	0.72 (0.09)	0.284	5.31 (0.11)	0.001
**Upper Midwest**	5.00 (0.10)	< 0.001*	1.53 (0.13)	1.00	5.58 (0.15)	< 0.001*
**Northern Rockies**	5.11 (0.23)	0.354	0.53 (0.28)	1.00	7.29 (0.34)	< 0.001*
**Southwest**	6.40 (0.12)	0.003	1.04 (0.15)	1.00	4.93 (0.18)	1.00
**West**	4.11 (0.08)	< 0.001*	1.27 (0.10)	1.00	2.86 (0.12)	< 0.001*
**Northwest**	6.02 (0.15)	1.00	1.47 (0.18)	1.00	4.41 (0.22)	1.00

* Denotes statistical significance

**Table 3:** Beta and p values from multiple linear regression models measuring the association of percent reported hearing loss, Audiologists per 10,000, Beale codes, and region (Appalachian vs. non-Appalachian).

	β	p-Value
**Model 4: Percent reported hearing loss**		
Beale Code	0.348	<0.001^*^
Appalachia	0.938	<0.001^*^
**Model 5: Audiologists per 10,000**		
Beale Code	–0.151	<0.001^*^
% Reported Hearing Loss	–0.147	<0.001^*^
